# Comparative Methods for Association Studies: A Case Study on Metabolite Variation in a *Brassica rapa* Core Collection

**DOI:** 10.1371/journal.pone.0019624

**Published:** 2011-05-13

**Authors:** Dunia Pino Del Carpio, Ram Kumar Basnet, Ric C. H. De Vos, Chris Maliepaard, Maria João Paulo, Guusje Bonnema

**Affiliations:** 1 Laboratory of Plant Breeding, Wageningen University, Wageningen, The Netherlands; 2 Plant Research International, Wageningen University and Research Centre (Wageningen-UR), Wageningen, The Netherlands; 3 Centre for BioSystems Genomics, Wageningen, The Netherlands; 4 Biometris-Applied Statistics, Wageningen University and Research Center, Wageningen, The Netherlands; University of Umeå, Sweden

## Abstract

**Background:**

Association mapping is a statistical approach combining phenotypic traits and genetic diversity in natural populations with the goal of correlating the variation present at phenotypic and allelic levels. It is essential to separate the true effect of genetic variation from other confounding factors, such as adaptation to different uses and geographical locations. The rapid availability of large datasets makes it necessary to explore statistical methods that can be computationally less intensive and more flexible for data exploration.

**Methodology/Principal Findings:**

A core collection of 168 *Brassica rapa* accessions of different morphotypes and origins was explored to find genetic association between markers and metabolites: tocopherols, carotenoids, chlorophylls and folate. A widely used linear model with modifications to account for population structure and kinship was followed for association mapping. In addition, a machine learning algorithm called Random Forest (RF) was used as a comparison. Comparison of results across methods resulted in the selection of a set of significant markers as promising candidates for further work. This set of markers associated to the metabolites can potentially be applied for the selection of genotypes with elevated levels of these metabolites.

**Conclusions/Significance:**

The incorporation of the kinship correction into the association model did not reduce the number of significantly associated markers. However incorporation of the STRUCTURE correction (Q matrix) in the linear regression model greatly reduced the number of significantly associated markers. Additionally, our results demonstrate that RF is an interesting complementary method with added value in association studies in plants, which is illustrated by the overlap in markers identified using RF and a linear mixed model with correction for kinship and population structure. Several markers that were selected in RF and in the models with correction for kinship, but not for population structure, were also identified as QTLs in two bi-parental DH populations.

## Introduction

In plants association mapping has been developed as a tool to relate genetic diversity, expressed as allelic polymorphisms, to the observed phenotypic variation in complex traits without the need to develop mapping populations. Results obtained with association mapping methods in various crops indicate that this technique can be successful in the identification of markers linked to genes and/or genomic regions associated to a desirable trait [1]–[6].

However, one of the most important constraints in the use of association mapping in crop plants is unidentified population sub-structure, which arises as a result of adaptation, genetic drift, domestication or selection [3], [7]. Spurious associations due to population structure may lead to false positive associations, if the cause of the correlation is not tight genetic linkage between polymorphic locus and the locus involved in the trait, but disproportional representation of the trait in one subpopulation [8].

As a consequence, when association mapping is used to identify genes responsible for quantitative variation in a group of accessions, there is enough evidence that confounding will be a significant problem, especially if the trait varies geographically, as is the case for example of flowering time [3], [9], [10].

Several methods can be used to infer multiple levels of relatedness in a population [10], [11]. The STRUCTURE program uses a Bayesian approach to cluster accessions of a collection into subpopulations on the basis of multilocus genotype data [12]–[14]. Designed statistical tests using PCA have also been used to check the existence of population structure in a data set and monitor the number of significant principal component axes [15]–[17]. Similarly, kinship coefficients approximate identity by descent between pairs of accessions. In several association studies information about population structure and/or kinship has been included into the general linear regression and mixed linear models [6], [10], [12], [18]. Results obtained in some studies suggest that the method that accounts both for subpopulations and kinship (also called the “QK method”) is the most appropriate for association mapping [10]. A different statistical approach, which carries one or more advantages above most other methods, is the Random Forest [19]. This is a tree-based method, that has been used for marker trait associations with human disease data, because it allows the ranking and selection among very large sets of predictor variables (markers) that best explain the phenotype [20], [21]. This method is computationally very fast, scale-free and makes no strong assumptions about the distribution of the data. For emerging types of datasets like metabolite profiles, transcript profiles and the very large SNP datasets that emerge due to the rapid development of whole genome sequencing technology, it is necessary to consider and validate association methods that can handle these high dimensional data sets.

Furthermore, the power to detect epistasis in moderately sized populations in general is low, while Random Forest can implicitly use interactions among regressor variables to predict the phenotype and can help identify multi-locus epistatic interactions [22], [23].

For this study we choose to work with a core collection of 168 *Brassica rapa* accessions, representing the wide variation in crop types (hereafter called morphotypes) and geographical origins. *Brassica rapa* has been cultivated for many centuries in different parts of the world, increasing the variation within the species as a result of breeding. *B.rapa* is a diploid species which includes vegetable-, fodder-and oil crops. The leafy vegetables include both heading types (Chinese cabbage) and non-heading types (among others Pakchoi, mizuna, mibuna, komatsuna and broccoletti, consumed for its inflorescenses), the turnips include vegetable and fodder turnips, and the oil crops include both annual and biannual crops. Most leafy vegetables, turnips and biannual oil types are self-incompatible and as a consequence the genebank accessions of this type are heterogeneous and plants are heterozygous. A smaller group of *Brassica rapa* is formed by the sarsons (brown sarson (*Dichotoma*), toria (*Dichotoma*) and yellow sarson (*trilocularis*)) characterized by very early flowering and self-compatibility of many accessions, which results in heterogeneous accessions with merely homozygous plants [24]. Modern cultivars and breeding lines from seed companies are homogeneous heterozygous hybrids and homozygous inbred lines.

In a previous study the genotypic fingerprinting of a large collection of 160 accessions showed that there is considerable genotypic variation within the *B. rapa* gene pool [24]. The hierarchical cluster analysis revealed that accessions from the same geographical region (Europe, Asia and India) are more related to each other genetically than accessions representing similar morphotypes from different geographical regions. These accessions from the same origin are genetically related possibly because they share part of their breeding history [24].

Previously, in a collection of 160 *B. rapa* accessions association analysis with correction for population structure led to the identification of 27 AFLP markers, related to the variation in leaf and seed metabolites as well as morphological traits [6]. In the present study we consider the genetic association between markers and tocopherols, carotenoids, chlorophylls and folate in a core collection of 168 *B. rapa* accessions of different morphotypes and origin. We explore the results obtained with association methods that correct for kinship and population structure which mainly aim to reduce the rate of false-positive associations, and in addition we make use of Random Forest for comparison to the commonly used association methods.

## Results

### Principal coordinate analysis (PCO) and population structure of the core collection

The genetic population structure of the core collection of 168 accessions was inferred using 553 markers (AFLP, *Myb* and SSR polymorphic bands).

**Figure 1:**
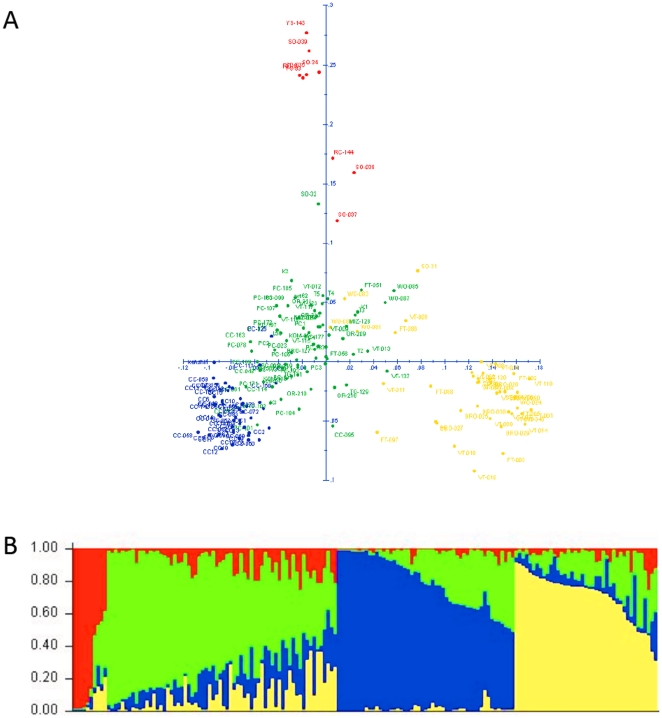
Principal coordinate analysis (A) and STRUCTURE (B) results. Colors define subpopulations: red (oil: Population 1), green (PC+T: population 2), blue(CC: population 3) and yellow (VT+FT: population 4).

The Bayesian clustering method as implemented in STRUCTURE revealed 4 subpopulations. Subpopulation 1 included oil types of Indian origin, spring oil (SO), yellow sarson (YS) and rapid cycling (RC) (SO, YS and RC); subpopulation 2 included several types from Asian origin: pak choi (PC), winter oil, mizuna, mibuna, komasuna, turnip green, oil rape and Asian turnip (PC+T); subpopulation 3 included mainly accessions of Chinese cabbage (CC) and subpopulation 4 included mostly vegetable turnip (VT), fodder turnip (FT) and broccoletto accessions from European origin (VT+FT) (Figure 1B).

There was a high level of admixture between the different subpopulations. Of the 168 accessions, 109 were assigned to a subpopulation with a membership probability of p>0.70. Fifty-nine accessions were assigned to more than one subpopulation and had membership probabilities below 0.7 corresponding to several subpopulations (Table S1).

The PCO-MC is a method, which couples principal coordinate analysis to a clustering procedure for the inference of population structure from multi-locus genotype data. The PCO and STRUCTURE output produced comparable results. After the PCO analysis, in the second dimension one small distinct, statistically significant subpopulation, corresponding to oil types of Indian origin, could be distinguished. This subpopulation corresponds to subpopulation 1 (SO, YS and RC) as identified in STRUCTURE (Figure 1A). In the first dimension, the three subpopulations as defined in STRUCTURE form three clusters with overlap. On the right, the cluster of yellow dots corresponds to accessions in subpopulation 4 (VT and FT from Europe) as defined in STRUCTURE, and on the left the blue dots represent the accessions corresponding to subpopulation 3 (CC), while the green dots represent accessions that correspond to subpopulation 2 (PC and T from Asia). When the top 5 components are calculated, they together account for 30% of the total variation present in the core collection. As many principal component loadings would have been needed to account for the variation within this collection, we decided to include STRUCTURE output into the association model to correct for population structure.

**Figure 2:**
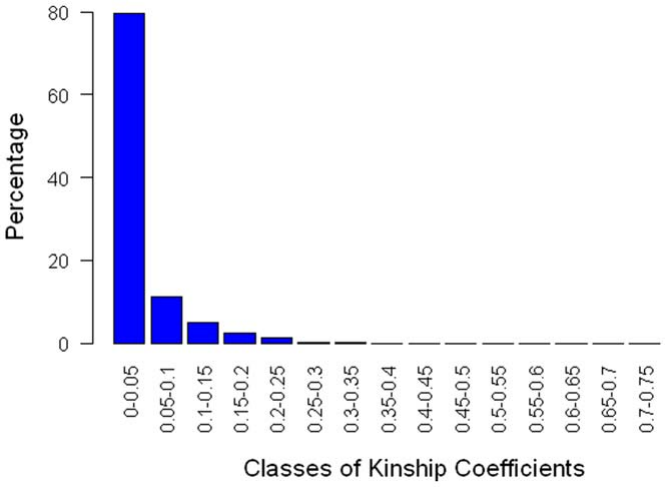
Distribution of kinship coefficients among 168 accessions of the *B. rapa* core collection.

In figure 2 we show the frequencies of the different kinship coefficient classes. The highest frequency was found for values between 0–0.05 (79.47%) while the second highest frequency was found for values between 0.05–0.1 (11.21%). These values are similar to the ones obtained in *Brassica napus* [25]in which the kinship calculation indicates a low level of relatedness between the accessions, with only few accessions being more related to each other.

### Metabolite variation

**Figure 3:**
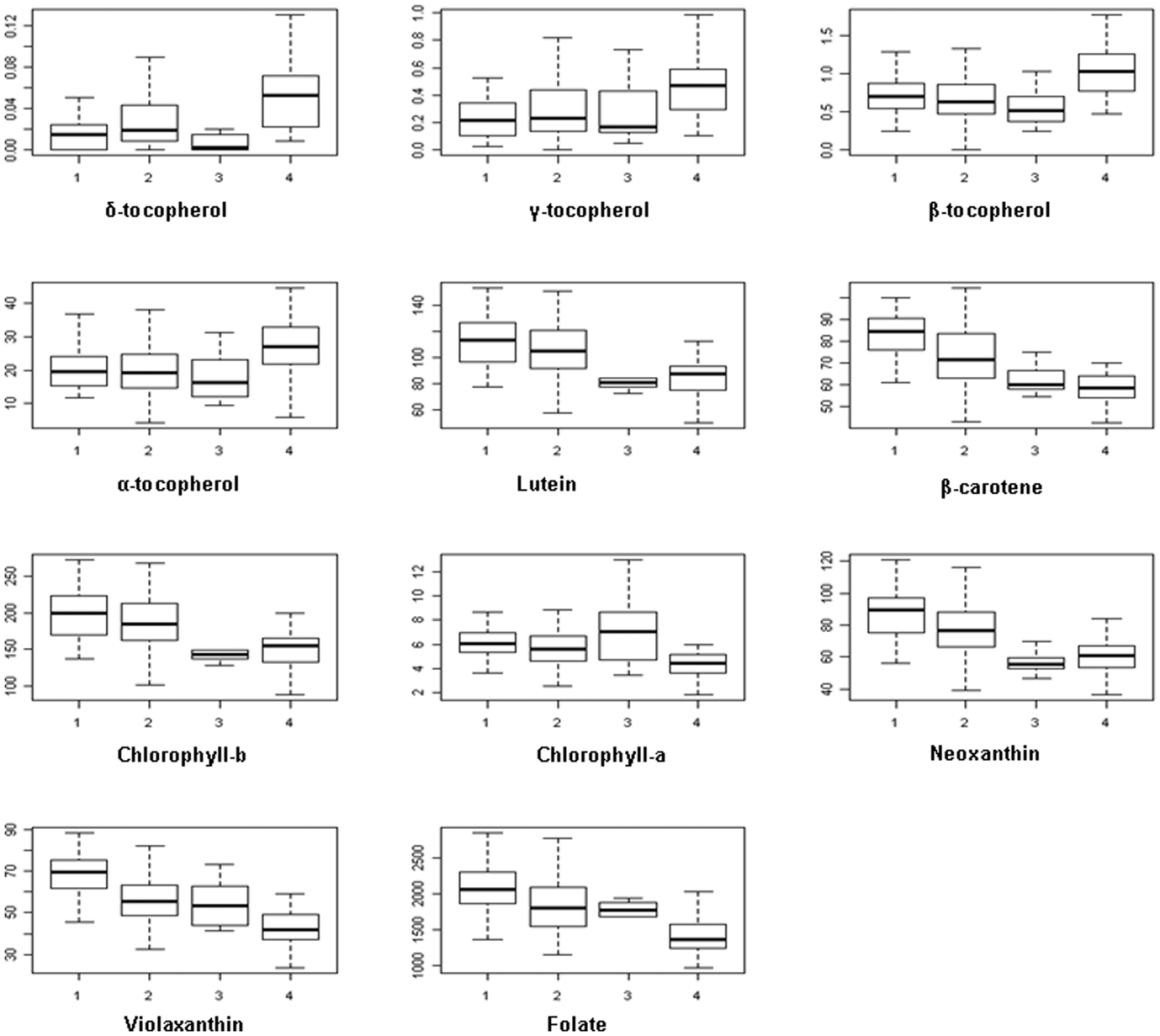
Boxplots of metabolite content variation present in sub-populations. The numbers indicate subpopulation as defined with STRUCTURE. Oil: Population 1, PC+T: population 2, CC: population 3 and VT+FT: population 4.

To estimate the variation within and between the different *B. rapa* morphotypes, boxplots were constructed based on the total content value per metabolite in each subpopulation as defined by STRUCTURE (Figure 3). Visual inspection of the box plots and the least significant differences (LSD) in metabolite content between subpopulations showed variation in the amount of most of the carotenoids and folate between these subpopulations. Conversely, the content of chorophyll *b* and lutein was significantly different between few subpopulations and the content of tocopherols was just significantly different between the Chinese cabbage (CC) subpopulation 3 compared to the other subpopulations (Table S2).

### Association analysis

#### Using linear and linear mixed models

Because many of the phenotypic trait values showed a distribution highly correlated to the underlying population structure it was expected that the number of significantly associated markers would differ to a large extent between different metabolites and between analysis methods, as shown in Table 1.

**Table 1:** Association mapping results from the different linear models and Random forests (RF) indicating the numbers of significant markers after correction for multiple testing (FDR P-value ≤0.05) for each metabolite.

	TOCOPHEROLS				CAROTENOIDS				CHLOROPHYLLS		
	δ tocopherol	γ tocopherol	β tocopherol	α tocopherol	Lutein	B-carotene	Neoxanthin	Violaxanthin	chlorophyll *b*	chlorophyll *a*	folate
Model (1)*	2	24	55	32	22	97	89	92	22	28	112
Model (2)	0	0	0	0	0	0	0	4	0	0	2
Model (3)	2	26	56	34	22	98	88	96	22	23	109
Model (4)	0	0	0	0	0	5	0	5	0	0	1
RF	16	24	12	8	16	36	39	34	32	17	28
RF-Model(4)**	0	0	0	0	0	3	0	3	0	0	1
RF-Model(1)**	1	11	4	2	6	31	26	30	9	7	21
RF-Model(3)**	1	11	4	2	6	32	26	30	9	7	21

*Model (1): naïve model; model (2) correction for Q; model (3) correction for K; model (4) correction for K and Q; RF Random Forsest.

**number of markers identified in both methods are listed.

To test for marker-trait associations we first applied an approach that did not include any correction for the level of relatedness or structure between accessions (model 1). As a result the number of significantly associated markers to a specific metabolite after multiple test correction was strongly inflated and ranged from 2 (for δ tocopherol) to 98 (for folate) per metabolite. The highest numbers of significant markers associated to a trait (>80) were found for β-carotene, neoxanthin, violaxanthin and folate; these metabolites also showed the greatest variation in content between subgroups.

To account for the level of relatedness between individuals we included the kinship correction (K matrix) in model (3). However, with the inclusion of this correction the number of significantly associated markers remained high (2–94). The results of these two models are highly similar not only in number but also in the identity of the significant markers for each metabolite.

In addition to the K matrix we introduced the STRUCTURE Q matrix as a correction. After accounting for population structure in model (2) the number of significant markers found per metabolite after a multiple correction step was dramatically reduced. Only for violaxanthin and folate few markers were identified. This drop down was as strong for the metabolites with subpopulation variation (carotenoids and folate) as for the tocopherols, which showed significant variation only between the CC subpopulation and the other subpopulations.

When we combined the information from the Q matrix and the K matrix in the full model (4), following the described approach [10], the performance is comparable to model (2), which includes the Q matrix only, in both the obtained number of associations and the identity of associated markers, except for β-carotene with five markers identified in model 4.

After correcting for multiple testing in the KQ correction model, only ten markers remained significantly associated with metabolites: Alu_M476_0, pTAmCAC_148_3, Hae_M294_2, pGGmCAA_335_2 and pTAmCAT_312_3 for β-carotene; Alu_263_6, pTAmCAC_101_7 and pTAmCAC_270_9, Br13 and Br46 for violaxanthin and pGGmCAA_335_2 for folate.

**Figure 4:**
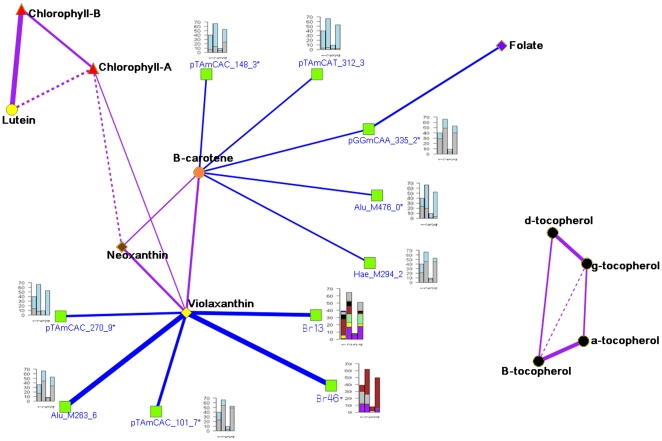
Network of partial correlation between metabolites, and marker-metabolite association under model 4 (KQ correction). The thicker the line, the stronger the correlation and or association. Shape and color of vertices indicate metabolites and associated markers: β-carotenoids – round-orange; chlorophylls – triangle-red; folate- diamond-purple; lutein- round-yellow; neoxanthin- diamond-brown; tocopherols- round-black; violaxanthin- diamond-yellow and associated markers (model-4)- square-green. The allele frequency distribution of each associated marker according to STRUCTURE sub-populations is illustrated with barplot. Colours in barplots represent different marker alleles. *- indicates markers that are common between model 4(KQ correction) and RF.

To summarize the results obtained from the full model (4), we constructed a network with a total of three *Myb*, five AFLP and two microsatellite markers significantly associated to the metabolites (P<0.05). This network allowed us to connect the metabolites of similar pathways through markers (Figure 4).

The overlap of significant associated markers between all the pathways (carotenoids, tocopherols, chlorophylls and folate) was very limited as expected if we consider that biochemically different precursors are involved. We found only one marker (pGGmCAA_335_2) that was significantly associated to folate and β -carotene.

#### Random Forest

The number of significantly associated markers per metabolite ranged from eight for α-tocopherol to 39 for neoxanthin. Interestingly, when compared to the simple model (1), the number of significant markers obtained with the RF approach was much lower for all the metabolites except for δ-tocopherol.

Nonetheless, the overlap of significant markers between methods is large; many of the significant markers found with RF were also significant with the simple model (1) and with the model with correction for kinship (3). For example, an overlap between 20% and 30% of significant markers was observed for β-carotene, neoxanthin, violaxanthin, folate, α-tocopherol and β-tocopherol (Table 1 and Table S3).

In contrast, when the results obtained with Random Forest are compared to the results obtained with the full model (4) six out of the ten markers from this model are included in the Random Forest output.

In the case of the microsatellite markers the overlap between significantly associated markers in Random Forest and in model (1) was high and almost complete for both markers except for those identified for δ-, β- and α-tocopherol. Additionally, one out of two significant SSR markers from model (4) were found also significant in the Random Forest output.

## Discussion

An important consideration for the use of association mapping in crop plants is the presence of population structure. If a group of diverse accessions is chosen for this type of studies the risk exists that some of the accessions are more closely related to each other than the average pair of individuals taken at random in a population [8]. In our study we identified with STRUCTURE the presence of 4 subpopulations, which showed correlation with the origin and morphotypes of *B. rapa*. Results from both principal coordinate analysis (PCO) and STRUCTURE illustrate the highly admixed nature of the accessions within this collection. We decided to use membership probabilities obtained from STRUCTURE in the association mapping model to correct for populations structure, as it is a widely used method. In addition correction for kinship was included in other models.

In the four models we explored the impact from STRUCTURE (model 2), kinship coefficients (model 3) or both (model 4) in the association models.

Correcting for the level of relatedness using the Q matrix from the STRUCTURE output, resulted in a significant reduction of the number of marker-trait associations as shown by comparing model (1) and both models (2) and (4). Although there was always some overlap between the marker-trait associations identified by these models, new associations arose with models (2) and (4).

The inclusion of the kinship matrix in models (3) and (4) did not reduce the number of significant marker-trait associations. This was most likely due to the fact that kinship values were very low and the accessions of the core collection showed similar levels of relatedness. The results from STRUCTURE and the identical levels of relatedness as observed in K seem to contradict. Similarities based on the Jaccard measure were also tested in model (3), with the same results as obtained with the similarities obtained from SPAGeDi.

We tested thereafter both corrections in phenotypic models identical to models (2) and (3) but without the marker effect, and compared the resulting residual variance with the “empty” model: trait  =  error. We found that whereas Q explained the phenotypic variation by as much as 60% for some traits, the K matrix did not seem to explain any part of the phenotypic variation, for all traits. This seems to support earlier evidence that K alone in some cases may not correct for population structure [25]. In terms of how these methods performed in reducing the false positive rate, we observed that metabolites with a distribution highly correlated to the underlying population structure, like for example the carotenoids, still retained the highest number of associated markers in all the statistical models. As a result, in spite of introducing a correcting term in our models we still expect some false positives within this list of significant markers. Even in association studies with Arabidopsis inbred lines it is difficult to distinguish true associations from false ones because of confounding by complex genetics and population structure [26].

In the present study we considered the use of Random Forests (RF) as a complementary method to our association study. The performance of this method in association analysis has been recently tested in Arabidopsis [27]. Within that study the overlap of RF and Fisher's exact test was considerable.

In our study we evaluated the RF results in comparison to the results obtained with the already validated and widely used model (4) and the simple model (1). One striking result of the RF analysis is the small number of associated markers that are found for all the metabolites in comparison to model (1). Random Forests is rather robust to outliers, as opposed to linear models, making it an attractive alternative to the traditional linear models. We decided to evaluate the overlap of RF and the simple model (1), which does not include any correction, and the full model, which includes the Q and K matrix correction (4). Seven out of eleven marker-trait associations found significant after multiple test correction with model (4) were also found significant with RF, while also many Random Forest markers were identified with models 1 and 3 (K correction).

Several markers that are associated with the metabolites studied, were also identified in QTL studies for the same metabolites in DH populations derived from crosses between two accessions (yellow sarson (YS 143) x pakchoi (PC 175) and their reciprocal cross (Table S3), or map to regions that harbour structural genes in the metabolic pathway based on Arabidopsis-*B. rapa* genome synteny (data not shown). This is a confirmation of the effect of the marker-trait association and makes these markers important candidate genes for further study.

For eight of the eleven metabolites analyzed, Random Forest selected at least one marker that mapped in the QTL interval for the respective metabolites in the biparental QTL studies. For several metabolites except the tocopherols, these markers were also identified in model 1 (no correction) and 3 (kinship correction) but not in models 2 and 4 (with Q correction).

In these same two doubled haploid populations QTL for lutein and chlorophyll-a and -b overlap in the region where the marker pTAmCAC_148_3 is located and identified as significant for β-carotene by all models. In this genomic region of linkage group A03 the genes ε-cyclase, β-carotene hydroxylase and carotenoid isomerase are predicted based on synteny with Arabidopsis [28] and represent potential candidate genes for β-carotene and lutein. In the case of violaxanthin the marker Alu_263_6 was identified as associated in model 4 (K and Q correction). Alu_263_6 is 5 cM apart from the structural gene Phytoene desaturase that we mapped in the biparental DH population. For most markers mappositions are not available, however the linked microsatellite marker Br13 and marker Alu_263_6 on A08, were both associated to violaxanthin.

In this study we have identified several markers that can be applied to screen *B. rapa* collections or breeding populations to identify genotypes with elevated levels of important metabolites that are considered as healthy compounds. While further validation of these markers for marker assisted selection in *B. rapa* is needed, at least the eight myb and AFLP markers and two microsatellites markers found significant with model (4), after multiple testing correction [29], and also with Random Forest, plus the markers identified using both Random Forest and the models (1) and (3) should be considered as likely candidates for further work.

At present we are in the process of expanding the core collection so that association mapping within the four subpopulations becomes feasible and to increase the power of the statistical analysis. In an attempt to separate true from spurious associations and/or false negatives in future association studies using the present core collection we will follow a similar approach, which takes into account the level of relatedness between individuals (K and Q) and the use of Random Forest.

## Materials and Methods

### Plant material

The *Brassica rapa* core collection included a total of 168 accessions of diverse morphotype and origin (Table S1).

The leafy vegetables, (Chinese cabbage, pakchoi and Japanese cultivars), neep greens, turnip rape, brocoletto (turnip tops) and turnip types are mainly self-incompatible and as a consequence the accessions are heterogeneous and heterozygous. The annual yellow sarson oil seed accessions are self-compatible, which results in homozygous plants. The modern cultivars and breeding lines from seed companies are homogeneous hybrids and inbred lines. 137 accessions were obtained from the Dutch Crop Genetic Resources Center (CGN) in Wageningen, the Chinese Academy of Agricultural Sciences (CAAS)-Institute for Vegetable and Flowers (IVF) and the CAAS Oil Crop Research Institute (OCRI) genebanks and the Osborn Lab, while six different breeding companies (Table S1) provided 31 accessions. For the metabolite profiling two plants per accession were sown in the greenhouse (2006) under the following conditions: 16 hours light and temperature fluctuation between 18 and 21°C. The plants were distributed over two tables in a randomized design with one plant per accession on each table. In the 5th week after transplanting the leaf material (youngest expanded leaves) was harvested per plant. Upon harvesting, all plant materials were snap-frozen in liquid nitrogen and ground into a fine powder using an IKA A11 grinder cooled with liquid nitrogen. Frozen powders were stored at −70°C until analyses. DNA was extracted from the ground and frozen material with the DNAeasy kit (Qiagen, USA).

### Metabolite analyses

#### Folate extraction and analysis

From each frozen powder, 0.15 g was weighed and 1.8 ml of Na-acetate buffer containing 1% ascorbic acid and 20 µM DTT, pH 4.7, was added. After sonication for 5 min and heating at 100°C for 10 min, total folate content of samples was quantified using a Lactobacillus casei–based microbiological assay, after enzymatic deconjugation for 4 h at 37°C pH 4.8, with human plasma as a source of γ-glutamyl hydrolase activity [30]. Each extract was assayed in 4–6 replicates using different dilutions. The total technical variation of this analysis was determined using 7 replicate extractions from the same frozen powder of two different randomly chosen genotypes, and was 5.5% and 6.9%, respectively.

#### HPLC analyses of lipid-soluble phytonutrients

Extraction and analyses of carotenoids, tocopherols and chlorophylls were performed as described in Bino et al. [31]. In short, 0.5 g of FW of frozen powder was taken and extracted with methanol-chloroform-Tris buffer twice, the chloroform fraction was dried using nitrogen gas and taken up in 1 ml of ethylacetate. The chromatographic system consisted of a W600 pump system, a 996 PDA detector and a 2475 fluorescence detector (Waters Chromatography), and an YMC-Pack reverse-phase C30 column (250×4.6 mm, particle size 5 µm) at 40°C was used to separate the compounds present in the extracts. Data were analyzed using Empower Pro software (Waters Chromatography). Quantification of compounds was based on calibration curves constructed from respective standards. The total technical variation was between 2 and 8 percent, depending on compound, as was established using 12 extractions of the same frozen powder from a randomly chosen genotype.

### Genotypic data

The AFLP procedure was performed as described by de Vos et al.[32]. Total genomic DNA (200 ng) was digested with two restriction enzymes *Pst I* and *Mse I* and ligated to adaptors. Pre amplifications were performed in 20 µl volume of 1x PCR buffer, 0.2 mM dNTPs, 30 ng of adaptor primer, 0.4 Taq polymerase and 5 µl of a 10x diluted restriction ligation mix, using 24 cycles of 94°C for 30 s, 56°C for 30 s and 72°C for 60 s. Pre-amplifications products were used as template for selective amplification with three primer combinations (P23M48, P23M50 and P21M47).

For the *Myb* family targeted profiling, total genomic DNA was digested using the following enzymes per reaction: Hae III, Rsa I, Alu I and Mse I and ligated to an adaptor. Pre amplifications with one primer directed to a common *myb* motif (Dr. Gerard van der Linden, Wageningen UR Plant Breeding , unpublished results) and one adaptor primer were performed in 25 µl of 1X PCR buffer (with 15 Mm MgCl2), 0.2 mM dNTPs, 0.8 pMol Gene specific primer, 0.8 pMol Adapter primer, U Hotstar Taq polymerase (Qiagen) and 5 µl of a 10X diluted restriction ligation mix. Amplification products were used as template for selective amplification.

AFLP and *Myb* profiling images were analyzed using Quantar Pro™ software. This marker dataset (359 polymorphic bands) was scored as present (1) or absent (0) and treated as dominant markers. A map position could be assigned for 69 markers from this dataset; these markers were distributed over different positions in the linkage groups of a doubled haploid population (Pino Del Carpio, unpublished results).

For microsatellite (SSR) screening, 28 primers were selected for amplification in the accessions of the core collection. From the primers 10 were genomic and 18 were new Est based SSRs (Dr. Ma RongCai, Dr Tang Jifeng (WUR-PBR)). The primers were selected because of their map position in different maps of *B. rapa* and distribution over all the linkage groups (A01–A10) [33]. Microsatellites scores were converted to binary data per observed allele (194 fragments of defined size) as present (1) or absent (0) and were also treated as dominant markers.

### Assessment of population structure

Marker data (AFLP, *Myb*, SSR) were used to identify the different subgroups and admixture within the accessions of the core collection through a model of Bayesian clustering for inferring population structure. For the SSRs only the most frequent SSR allele was taken into account to avoid over representation of the SSR loci.

A total of 539 markers was included in the analysis, and ploidy was set to one. The number of subpopulations was determined using the software STRUCTURE 2.2 (http://pritch.bsd.uchicago.edu/software), by varying the assumed number of subpopulations between one and ten, with a total of 300,000 iterations for Markov Chain Monte Carlo repetitions and 100,000 burns in.

In addition, we also followed the procedure PCO-MC as described in [16], to assess population structure. The method uses principal coordinate analysis (PCO) and clustering methods to infer subpopulations in a collection of accessions. We chose this method to complement the analysis performed by STRUCTURE because it is computationally efficient and model free and has been shown to be capable of capturing subtle population structure [16]. We used software NTSYS version 2.2 [34] to produce pairwise distances, among all accessions, based on the Jaccard measure. Principal coordinates were obtained based on the distance matrix as described by Reeves and Ritchards [16]. Then the procedure PROC MODECLUS in SAS 9.1 software (SAS Institute, Cary, NC) was used to group the accessions into subpopulations according to kernel density estimates in the PCO space. Subpopulations were formed by decreasing order of the kernel densities, starting with the largest estimated kernel density (by setting method = 6 at PROC MODECLUS). We performed a test to determine which subpopulations were significantly distinct from the rest, using PROC MODECLUS, and estimated stability values for the subpopulations using the PCO MC software.

(http://lamar.colostate.edu/~reevesp/PCOMC/PCOMC.html) [16]. The PCO plot of the first two components was drawn in DARwin software version 5.0.155 [35].

### Summary statistics of metabolite variation

Box plots were chosen as a tool to explore the variation of metabolite concentrations according to different STRUCTURE subpopulations. One-way ANOVA was performed for each metabolite to find the mean differences among the four STRUCTURE subpopulations. Least significant differences (LSD) were calculated to compare the differences of means of metabolite content between the four subpopulations obtained with STRUCTURE. Boxplots, ANOVA and LSD calculations were performed using R statistical software.

### Association analysis

Association analysis was performed in several steps of increasing complexity; with and without correction for population structure [10] using TASSEL (www.maizegenetics.net). A total of 243 markers with an allelic frequency higher than 10% was included in the association analysis. Since AFLP and *Myb* markers gave dominant marker scores and TASSEL works with co-dominant data, within TASSEL we set the ploidy to one to work with dominant scores as we had done with STRUCTURE. In the case of the 28 microsatellites all alleles were included within TASSEL in a different run as codominant markers.

In the first step a “naïve” model was used to associate each marker to the trait, 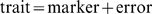
(1)This model was fitted by a least squares fixed effects linear model in TASSEL where the markers are considered as a factor taking the value 0 (fragment absent) or 1 (fragment present). In this case a t-test could also have been used to test association since we only have two classes for the marker. In this “naïve” model population substructure was not taken into account.

In the second step the vector of cluster memberships Q obtained from Structure was added as a fixed term to the previous model
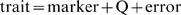
(2)In the third step we corrected for kinship using a linear mixed model available in TASSEL. The model can be written as 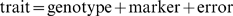
(3)where random terms are underlined. Genotype is a random factor with the different genotypes or accessions in the population. Kinship coefficients were calculated using SPAGeDi [36]. Like for the calculation of STRUCTURE, for the SSRs only the most frequent SSR allele was taken into account to avoid over representation of the SSR loci. We have V_G_ = σ^2^K; V_G_ is the variance-covariance matrix of the random genotype effects, K is the matrix of kinship coefficients and σ^2^ is the additive genetic variance.

In the fourth and final step we correct for kinship as well as population structure using a linear mixed model that combines the information contained in the two previous models. It is also known as the Q+K method [10].

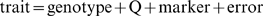
(4)As before, genotype is a random factor, with covariances given by the kinship matrix K and Q is a fixed term containing the cluster memberships. The model is similar to that described by Yu et al. [10] and Malosetti et al. [18]. Here we used the same set of AFLP, *Myb* and SSRs data to estimate both K and Q. The percentage of variation was also implemented in TASSEL and extracted from the output for further analysis and comparison.

### Correction for multiple testing

The p-values resulting from all the models for association analysis were corrected for multiple testing using a resampling method as implemented in the R package “multtest” [37].

### Random Forest

Random Forest (RF) regression [19] was used in this study to find markers (among the 243 AFLP and *Myb*, and 28 SSR marker set) associated to the tocopherol, carotenoids, flavonoids and folate metabolites. This method uses a bagging approach by bootstrapping samples [38] and gives the relative importance of each marker in the regression of metabolites. In this study, RF was performed using 5,000 regression trees for each analysis. Each tree is formed on a bootstrap sample of the individuals (training dataset), while individuals that are not in the bootstrap sample (out-of-bag samples =  OOB), are used for estimation of the mean squared error of prediction. Within each regression tree, at each split of the tree, a random subset of the markers is considered as a candidate set of markers for a binary split among the set of individuals. The partitioning of the samples is continued until homogeneous groups of small number of samples remain.

This procedure is fast and can handle high-dimensional data (predictor variables >> number of samples). Each tree is fully grown (unpruned) to obtain low-bias, high variance (before averaging) and low correlation among trees. Finally, RF averages are calculated over all the trees and result in low bias and low variance of predictions of the trait based on the markers used in the Random Forest [39] . This method has an internal cross-validation (using the OOB samples) and has only a few tuning parameters which, if chosen reasonably, do not change results strongly [38].

The parameter “mtry”, which indicates the number of random variables considered at each splitting node, was optimized by choosing the “mtry” with the highest percentage of explained variation among separate RF analyses done on “mtry” values 3, 6, 12, 24, 48 and 96 successively on the same data set. The variance explained in RF is defined as 1-(Mean square error (MSE) / Variance of response), where MSE is the sum of squared residuals on the OOB samples divided by the OOB sample size [40]. The “mean decrease in MSE” (InMSE) was considered to quantify the importance of each marker. The higher the “InMSE” value of the marker, the greater the increase in explained variation when it is included in the model.

In general RF yields only the relative importance of markers that explain the variation present in metabolites, but does not give a significance threshold level to select a subset of associated markers. Therefore, a permutation method was used to calculate the significance of each marker association in this study [41]. All the observations of a metabolite (the response in the regression) were permuted to destroy the association between markers and metabolite, and RF analyses were repeatedly conducted on the permuted metabolite data 1000 times. For each metabolite, the “IncMSE” values of each marker from 1000 RF runs on permuted metabolites were stored, and uses as a “null distribution” of the IncMSE value to assess the significance threshold of each marker. Then, the IncMSE values of each marker obtained from RF analysis on the original unpermuted metabolite data set was compared to this “null distribution” at 0.05 level of significance to determine significantly associated markers.

RF regressions of metabolites on markers were conducted using the “Random Forest” package of the R-software [42].

### Network visualization of metabolite and marker correlation

A network is an extended graph, which contains additional information on the vertices and edges of the graph [43]. We used full-order partial correlation coefficients to construct correlation network of metabolites to remove the correlation between metabolites due to direct and indirect dependencies on the up-stream metabolites in the pathway. We included in the network graph all the markers that were associated to the metabolites after correction for multiple testing (α = 0.05). Since we are focusing on the tocopherols, carotenoids and folate pathway, correlation analysis can give spurious correlation between the metabolites due to the effect of upstream metabolites of the pathway. Partial correlation measures only the direct or unique parts of relation between metabolites controlling the effects of other metabolites of the pathway [44]. The only significant non-zero pairwise partial correlation coefficients (α = 0.05) between metabolites were shown in network. The vertices of the network are the metabolites, in this case tocopherols, carotenoids, chlorophylls and folate, and associated markers, whereas the edges correspond to metabolite-metabolite partial correlations and marker-metabolite association. For the visualization of the marker-metabolites association, the P-values obtained from model (4) were transformed into –log10(P-value). The network was constructed using the Pajek graph drawing software [45].

## Supporting Information

Table S1 **Membership probabilities and group assignment of all accessions used in this study based on STRUCTURE.**
(XLSX)

Table S2 **LSD result of metabolite variation based on STRUCTURE subgroups**
(XLSX)

Table S3 **Overview of associations between markers and metabolites for all metabolites investigated in this study across methods with p-values after multiple testing correction.** For mapped markers genetic map positions are listed. For RF only significant markers are indicated. In the last column QTL identified in DH populations from crosses between YS 143 and PC 175 and their reciprocal cross (map presented in [46]) are listed.
(XLSX)
